# Two Half-Sandwiched Ruthenium (II) Compounds Containing 5-Fluorouracil Derivatives: Synthesis and Study of DNA Intercalation

**DOI:** 10.1371/journal.pone.0120211

**Published:** 2015-03-19

**Authors:** Zhao-Jun Li, Yong Hou, Da-An Qin, Zhi-Min Jin, Mao-Lin Hu

**Affiliations:** 1 Institute of Agricultural Resources and Regional Planning, Chinese Academy of Agricultural Sciences, Key Laboratory of Plant Nutrition and Fertilizer, Ministry of Agriculture, Beijing, China; 2 Institute of Biotechnology and Nucleic Technology, Sichuan Academy of Agricultural Sciences, Chengdu, China; 3 College of Chemistry and Materials Engineering, Wenzhou University, Wenzhou, China; 4 College of Pharmaceutical Sciences, Zhejiang University of Technology, Hangzhou, China; University of Quebect at Trois-Rivieres, CANADA

## Abstract

Two novel coordination compounds of half-sandwiched ruthenium(II) containing 2-(5-fluorouracil)-yl-N-(pyridyl)-acetamide were synthesized, and their intercalation binding modes with calf thymus DNA were revealed by hyperchromism of ultraviolet-visible spectroscopy; the binding constants were determined according to a *Langmuir* adsorption equation that was deduced on the base of careful cyclic voltammetry measurements. The two compounds exhibited DNA intercalation binding activities with the binding constants of 1.13×10^6^ M^-1^ and 5.35 ×10^5^ M^-1^, respectively.

## Introduction

Cisplatin shows a strong ability of binding to and causing crosslinking of DNA in *vivo*, which ultimately triggers cell apoptosis, and is used successfully to treat some special types of cancers. The discovery of cisplatin has aroused great effort toward the design of metal-based anticancer drugs [1]. Particular interest is attracted toward ruthenium element, a member of platinum-group, due to its low toxity and its ability to mimic iron in binding with proteins in the plasma [2]. In the recent decades, as a potential anticancer medicine, organometallic ruthenium compounds of the type [Ru(*η* ^*6*^-arene)] are under intensive investigation, owing to their promising activity in *vitro* and in *vivo* toward cancer cell, including cisplatin-resistant cells [3].

Combining two or more multifunctionalities into one is a popular strategy in design new therapeutic agents. 5-Fluorouracil (5-FU) has been used extensively in the treatment of solid tumors for years, and in the late, its strong toxicities to the gastric system, intestinal mucosa and bone marrow is evidenced eventually. Therefore, attempts are made to improve its anticancer activity and to minimize its side effects by exploiting several prodrug, such as amines, alcohols, and peptides [4]. In the literature, covalent compound containing platinum(II) and 5-FU fragment has been reported [5], but covalent compound containing ruthenium and 5-FU fragment is not available.

The interaction of DNA with small molecule had been a hot subject of research [6–11] for long time, due to the interaction modes and kinetic mechanism reflected various senses for developing molecular drugs and probers to monitor DNA structure [12–15]. In this respect, we synthesized two novel half-sandwiched ruthenium(II) compounds from dichloro(p-cymene) ruthenium(II) dimer and 2-[(5-fluorouracil)-yl]-N-pyridyl-acetamide for the first time, and studied the interaction of calf thymus DNA (CT-DNA) with them by cyclic voltammetry and UV-vis spectrometry.

## Experimental

### 1. Apparatus and reagents

CT-DNA with high molecular weight was extracted and purified by a method described elsewhere [16]. All other chemicals, including dichloro(p-cymene)ruthenium(II) dimer {[(*η* ^6^-*p*-cymene)RuCl_2_]_2_} and 5-FU, were of analytical reagent grade, and were purchased from sigma-aldrich Co. (USA).

The CT-DNA solution was prepared with 0.1 M KCl / 0.05 M Tris—HCl (pH 7.16) and stored at 4°C. The concentration of DNA was determined by UV absorbance at 260 nm, with the extinction coefficient (ε_260_) taken as 6600 M^–^1 cm^–1^.

The electrochemical measurements were performed on a CHI1030b electrochemical workstation (CH Instrumental, Chenhua Corp., Shanghai, China). Supporting electrolyte for all experiments was a 0.1 M KCl / 0.05 M Tris—HCl buffer solution, which was adjusted to pH 7.16 by HCl.

IR spectra were recorded on an EQUINOX-55 instrument (Bruker Optics, Germany). Elemental analysis was performed on a Perkin-Elmer 2400 CHNS/O elemental analyzer (USA). The UV-vis spectra were recorded at room temperature on a U-3010 spectrophotometer (Hitachi, Japan) equipped with 1.0 cm quartz cells. ^1^HNMR and ^13^CNMR spectra were recorded on a AVANCE-500 instrument using tetramethylsilane (TMS) as an internal standard at room temperature, and the chemical shifts were given in relative to TMS.

### 2. Synthesis of Ruthenium compounds I and II

**Fig 1:**
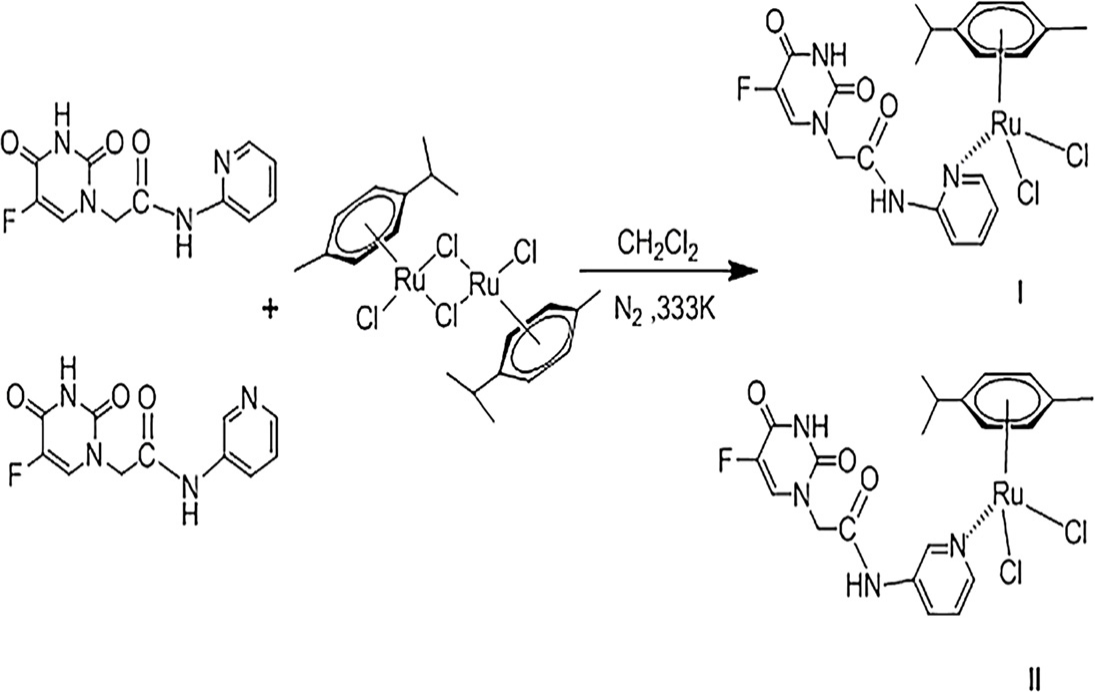
Synthetic routes of compound I and II.

Two isomeric 5-FU derivatives, 2-[5-fluoro-2,4-dioxo-3,4-dihydropyrimidin-1(2H)-yl]-N- (pyridin-2-yl)-acetamide (L1) and 2-[5-fluoro-2,4-dioxo-3,4-dihydropyrimidin-1(2H)-yl]-N-(pyridin- 3-yl)-acetamide (L2), were synthesized (Fig. 1) in accordance with a procedure described previously [17]. The two titled compounds were synthesized from the 5-FU derivative and dichloro(p-cymene)ruthenium(II) dimer. Typically, 2-[(5-fluorouracil)-yl]-N-pyridyl-acetamide (66 mg, 0.25 mmol) was added dropwise to a suspension of [(*η* ^6^-cymene)RuCl_2_]_2_ (76.5 mg, 0.125 mmol) in freshly distilled anhydrous methanol (25 mL). The color of the resulting mixture changed immediately with stirring at 25°C under argon, and precipitate was formed after storage in a freezer at -18°C for 24 h. The fine yellow solid was collected by filtration, recrystallized from methanol/ether, washed with methanol followed by ether, and dried overnight in vacuo.

$$\left[\left(\eta^{6}-p-\text{cymene}\right)\text{Ru}\left(\text{L1}\right){\text{Cl}}_{2}\right]$$ (I)

Yield based on [(*η* ^6^-cymene)RuCl_2_]_2_: 54.4%. IR: 3390 (υ N-H), 3291, 3044 (υ C-H), 2843 (υ-CH_2_-), 1705 (υ C = O), 1479 (υ C-N), 1430, 1375, 1234 (υ C-F), 879, 763, 686 cm^-1^ (S1 Fig.). Anal. calc. for C_20_H_23_Cl_2_FN_4_O_3_Ru (558.37), C 43.02% N 10.03% H 4.15%; found: C42.98% N 10.11% H 4.10%. ^1^HNMR (500 MHz, DMSO-*d*_*6*_) δ: 11.96 (1H, s), 8.11 (1H, d, *J* = 6.8 Hz), 7.90 (1H, dd, *J* = 5.1, 1.0 Hz), 7.43 (1H, ddd, *J* = 8.8, 7.2, 1.9 Hz), 6.51 (1H, d, *J* = 3.5 Hz), 6.49 (1H, d, *J* = 3.5 Hz), 5.84 (2H, d, *J* = 6.3 Hz), 5.80 (2H, d, *J* = 6.3 Hz), 4.36 (2H, s), 2.84 (1H, dt, *J* = 13.8, 6.9 Hz), 2.10 (3H, s), 1.20 (6H, d, *J* = 6.9 Hz) ppm (S2 Fig.). 13C NMR (126 MHz, DMSO-*d*_*6*_) δ: 169.49 (s), 159.10 (s), 157.47 (d, *J* = 25.8 Hz), 149.67 (s), 146.23 (s), 139.28 (d, *J* = 228.6 Hz), 137.78 (s), 111.77 (s), 108.62 (s), 106.37 (s), 100.09 (s), 85.95 (d, *J* = 107.1 Hz), 48.78 (s), 29.98 (s), 21.51 (s), 17.88 (s) ppm (S3 Fig.).

$$\left[\left(\eta^{6}-p-\text{cymene}\right)\text{Ru}\left(\text{L2}\right){\text{Cl}}_{2}\right]$$ (II)

Yield based on [(*η* ^6^-cymene)RuCl_2_]_2_: 61.3%. IR: 3520 (υ N-H), 3256, 3050 (υ C-H), 2926 (υ-CH_2_-), 1704 (υ C = O), 1485 (υ C-N), 1430, 1381, 1220 (υ C-F), 809, 694 cm^-1^ (S4 Fig.). Anal. calc. for C_20_H_23_Cl_2_FN_4_O_3_Ru (558.37), C 43.04% N 10.04% H 4.18%; found: C 43.02% N 10.03% H 4.15%. ^1^HNMR (500 MHz, DMSO-*d*_*6*_) δ: 11.95 (1H, d, *J* = 5.0 Hz), 10.54 (1H, s), 8.72 (1H, d, *J* = 2.2 Hz), 8.29 (1H, d, *J* = 3.6 Hz), 8.11 (1H, d, *J* = 6.7 Hz), 8.06–7.97 (1H, m), 7.37 (1H, dd, *J* = 8.3, 4.7 Hz), 5.82 (2H, d, *J* = 6.3 Hz), 5,78 (2H, d, *J* = 6.3 Hz), 4.53 (2H, s), 2.83 (1H, dt, *J* = 13.7, 6.9 Hz), 2.08 (3H, s), 1.19 (6H, d, *J* = 6.9 Hz) ppm (S5 Fig.). 13C NMR (126 MHz, DMSO-*d*_*6*_) δ:165.98 (s), 157.50 (d, *J* = 25.6 Hz), 149.77 (s), 144.60 (s), 140.43 (d, *J* = 64.6 Hz), 138.36 (s), 135.16 (s), 131.01 (d, *J* = 34.0 Hz), 126.14 (s), 123.77 (s), 106.38 (s), 100.08 (s), 85.92 (d, *J* = 106.7 Hz), 50.14 (s), 29.95 (s), 21.48 (s), 17.84 (s) ppm (S6 Fig.).

### 3. Preparation of (CT-DNA)-modified electrode

The electrodes were modified with CT-DNA in a procedure reported previously [18, 19]. Gold disk electrodes were polished with a series of alumina powder (1.0, 0.5 and 0.05 μm), then was purified and placed in fresh piranha solution (30% H_2_O_2_ and 70% H_2_SO_4_) to remove adsorbed organic impurities and sonicated in highly purified water for 5 min. Prior to the modification, the electrode surface was electrochemically activated by sweeping from -0.3 to +1.5 V in 0.1 M H_2_SO_4_ solution until a stable cyclic voltammogram characteristic of a clean gold electrode was obtained. After being washed with twice-distilled water, the freshly polished gold electrode was immediately modified by transferring a droplet of 10 μL of 1.0 μg μL^-1^ CT-DNA solution onto its surface, followed by air-drying overnight. The CT-DNA-modified electrode (CT-DNA/Au) was then soaked in sterile water for about 4 h and rinsed with water to remove unadsorbed CT-DNA.

### 4. Cyclic voltammetry

Voltammetric measurements were carried out in a conventional cell consisting of three-electrode, namely a bare gold (or CT-DNA/Au) as the working electrode, a saturated calomel electrode, and a platinum wire auxiliary electrode. A CT-DNA/Au electrode was soaked in buffer solution with different concentrations of the compound for voltammetric test designed to investigate the interaction of CT-DNA. Typical cyclic voltammetry experiment was carried out in a 5 mM solution of K_3_[Fe(CN)_6_] / K_4_[Fe(CN)_6_] (1:1) in the supporting electrolyte at room temperature (25°C). All solutions were deaerated with highly pure nitrogen, and the electrochemical experiments were performed at a scan rate of 0.1 Vs^-1^.

### 5. UV-visible spectra

The UV-vis spectrum was performed in 0.1 M KCl / 0.05 M Tris—HCl buffer at pH 7.16 and a temperature of 25°C, in the wavelength range of 200–600 nm. The concentration of CT-DNA was kept constant and the concentrations of two compounds were ranged from 0.04 mM to 0.20 mM.

## Results and Discussion

### 1. Electrochemical characterization of CT-DNA-modified electrode

**Fig 2:**
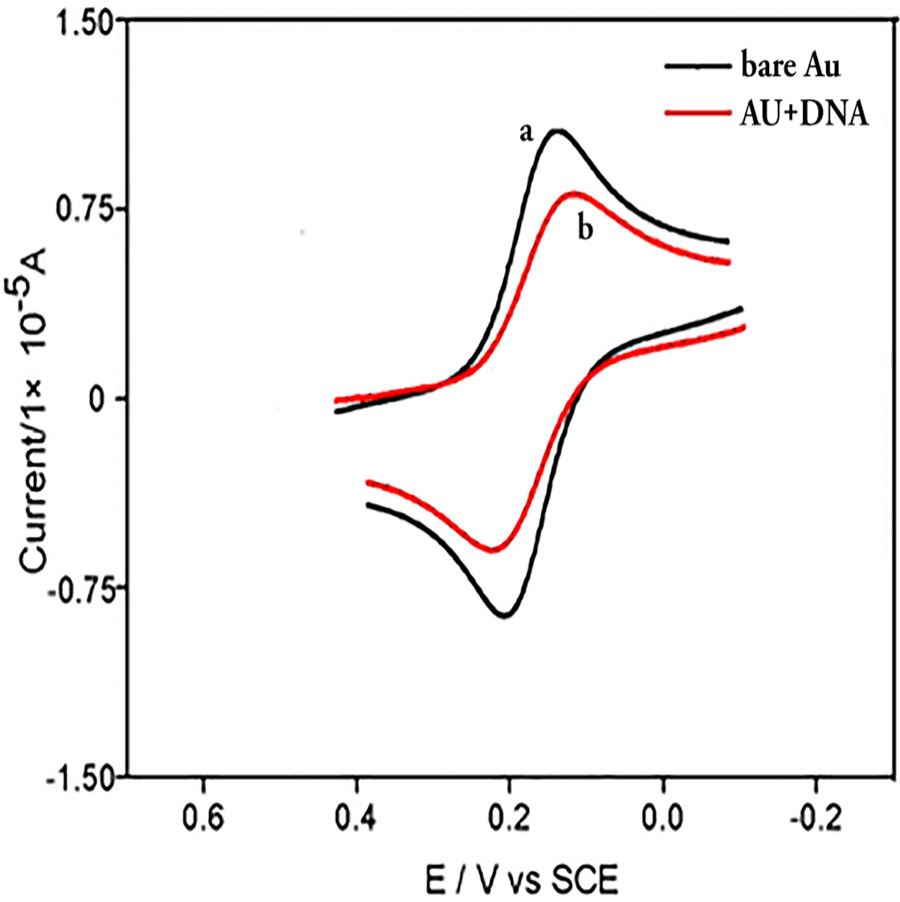
Cyclic voltammograms of Fe(CN)6^3-/4-^ in pH 7.16 Tris—HCl buffer solution at a bare Au electrode (a) and CT-DNA/Au (b), respectively. The scan rate was 0.1 V s^-1^ and the concentrations of Fe(CN)_6_^3-/4-^ and KCl were 5 mM.

Transition-metal compounds were often applied to probe both structural and functional aspects of DNA [20–21]. In this study, Fe(CN)_6_^3-/4-^ was used as an electrochemical probe molecule to characterize the interaction of DNA with the two titled compounds. As shown in Fig. 2, the cyclic voltammogram showed a couple of well-defined redox waves at bare gold with a peak-to-peak separation of 70 mV (curve a), and at CT-DNA/Au with such peak separation of 108 mV (curve b). Compared with curve *a*, the redox peak of curve *b* current decreased obviously. This indicated that CT-DNA had been assembled successfully on Au surface, and that CT-DNA acted as inert electron and mass transfer blocking layer and thus hindered the diffusion of ferricyanide towards the electrode surface. Further, the cyclic voltammogram of the same CT-DNA electrode remained stable after 20 scans in Tris-HCl buffer solution, suggesting high electrochemical stability of the DNA-coated film.

### 2. Interaction of the compounds with CT-DNA-modified gold electrode

**Fig 3:**
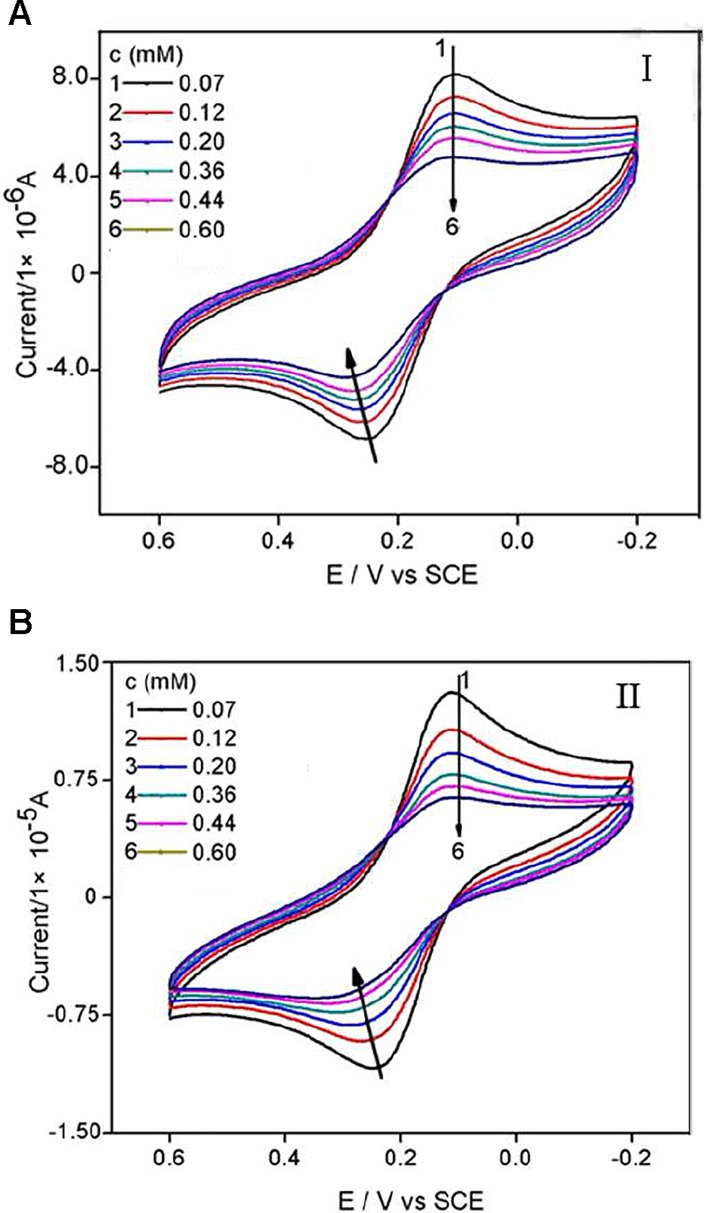
Cyclic voltammograms of Fe(CN)6^3-/4-^ in Tris—HCl buffer solution (pH 7.16) containing different concentrations of compound I and II. The scan rate was 0.1 V s-1 and the concentrations of Fe(CN)_6_^3-/4-^ and KCl were 5 mM.

**Fig 4:**
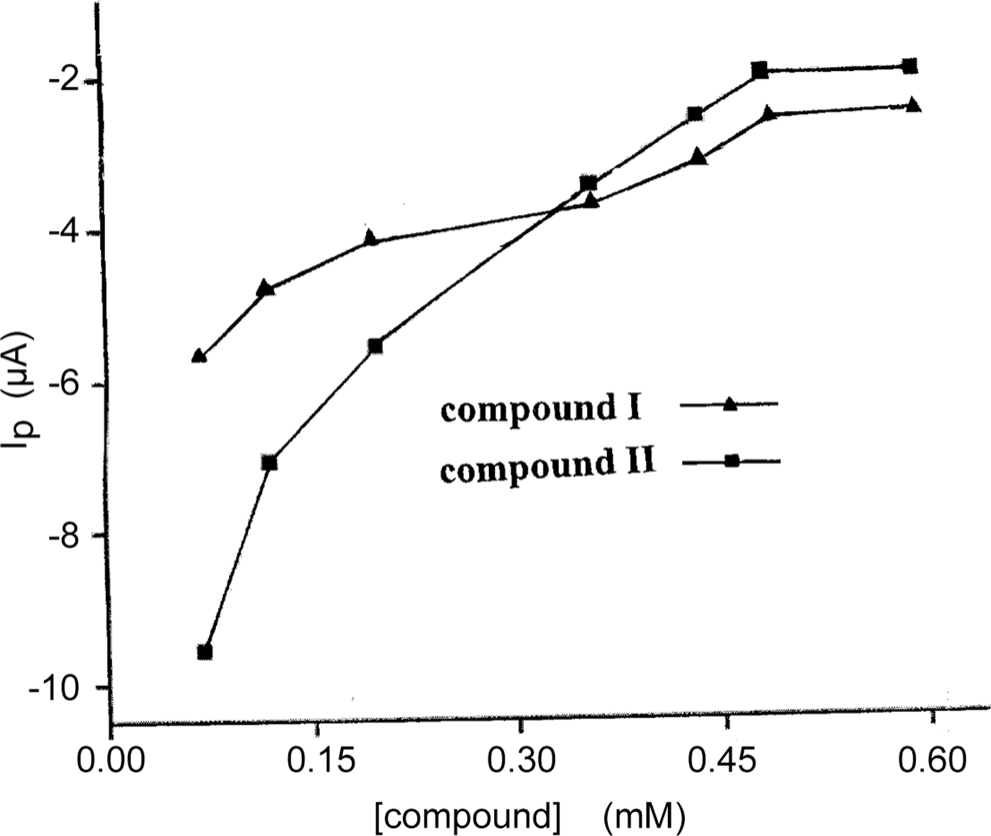
The dependence of decrease value of the peak current on the concentration of compound I and II.

As was shown in Fig. 3, the peak current of Fe(CN)_6_^3-/4-^ decreased as compounds (I or II) were added into the test solution. The more compound was added, the more the peak current of probe molecule decreased. As shown in Fig. 4, both peak currents of the cyclic voltammograms decreased with the concentrations of compounds increasing and tended to achieve a saturation value, i.e. at about 0.60 mM, as expected according to *Langmuir* adsorption behaviour. The reason might be attributed to that CT-DNA films made the redox process of Fe(CN)_6_^3-/4-^ at the gold electrode more difficult due to the physical blockage as well as possible electrostatic repulsion, and that the addition of the two compounds to the solution caused the CT-DNA film denser due to molecular interaction, therefore harder for Fe(CN)_6_^3-/4-^ ions to migrate through.

According to a previously reported method [22, 23], it was assumed that CT-DNA and DRUG produced a single complex of DNA·DRUG_m_ only.

$$\text{DNA + m DRUG }↔\text{ DNA}\cdot {\text{DRUG}}_{\text{m}}$$ (1)

The constant (*K*) was descripted as follows:

$$\text{K = }\frac{\left[\text{DNA}\cdot {\text{DRUG}}_{\text{m}}\right]}{\left[\text{DNA}\right]\cdot {\left[\text{DRUG}\right]}^{\text{m}}}$$ (1.1)

And the following equations could be deduced, where I indicated cyclic voltammetry current.

$$\Delta {\text{I}}_{\text{MAX }}=\text{k'}\cdot {\text{C}}_{\text{DNA}}$$ (1.2)

$$\text{and }\Delta \text{I}=\text{k'}\cdot \left[\text{DNA}\cdot {\text{DRUG}}_{\text{m}}\right]$$ (1.3)

$$\left[\text{DNA}\right]+\left[\text{DNA}\cdot {\text{DRUG}}_{\text{m}}\right]={\text{C}}_{\text{DNA}}$$ (1.4)

$$\Delta {\text{I}}_{\text{max}}-\Delta \text{I = k'}\left({\text{C}}_{\text{DNA}}-\left[\text{DNA}\cdot {\text{DRUG}}_{\text{m}}\right]\right)$$ (1.5)

$$\Delta {\text{I}}_{\text{max}}-\Delta \text{I=k'}\cdot \left[\text{DNA}\right]$$ (1.6)

As equations (1.3) and (1.6) were put into (1.1), it yielded:

$$\log\frac{\Delta \text{I}}{\Delta I_{\text{max}}-\Delta \text{I}}=\text{log K+m log}\left[\text{DRUG}\right]$$ (1.7)

$$\frac{1}{\text{ΔI}}=\frac{1}{\Delta {\text{I}}_{\text{max}}}+\frac{1}{\Delta {\text{I}}_{\text{max}}\cdot \text{K}}\cdot \frac{1}{{\left[\text{DRUG}\right]}^{\text{m}}}$$ (1.8)

**Fig 5:**
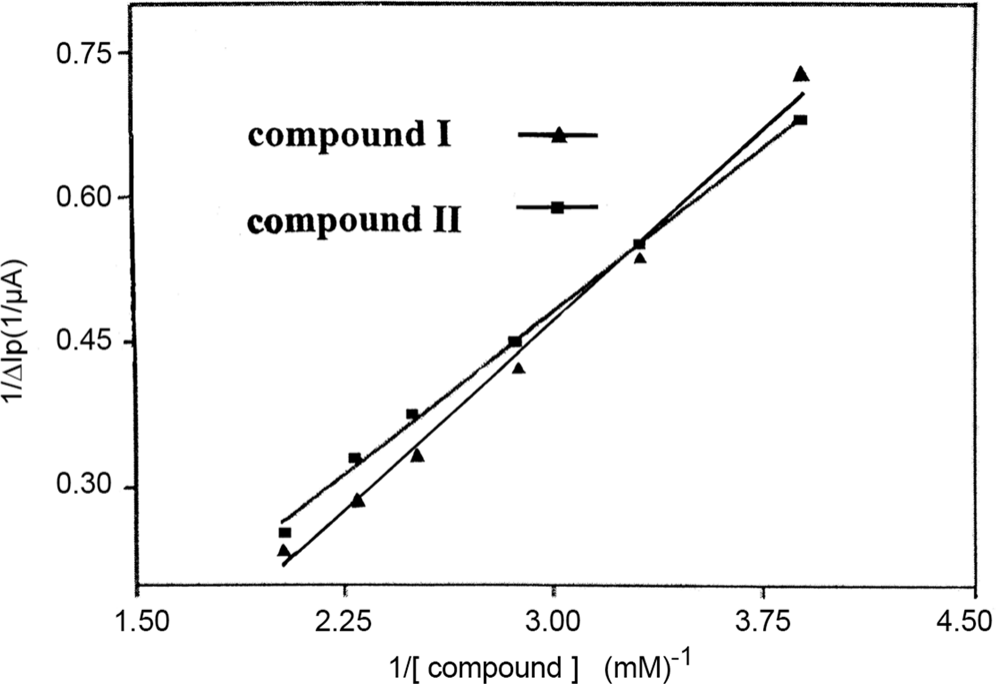
The relationship between 1/ΔI_P_ and 1/C.

As for Equation (1.8), we assumed *m* = 1, using ΔI_P_ to represent ΔI, and ΔI_p, max_ to represent ΔI_max_. As shown in Fig. 5, 1/[DRUG] demonstrated a good linear relationship with 1/ΔI_p_, indicating reasonable assumptive value of *m* for compounds I and II. Thus, the equation (1.9) was deduced [24], where ΔI_p_ = I_p_-I_po_, I_p_ and I_po_ represent the oxidation peak current of Fe(CN)_6_^3-/4-^ in the presence and absence of the drugs_,_ respectively; ΔI_p,max_ was the maximum difference of the oxidation peak current; and [DRUG] represented the concentration of the drug.

$$\frac{1}{\Delta {\text{I}}_{\text{P}}}=\frac{1}{\Delta {\text{I}}_{\text{p,max}}}+\frac{1}{\Delta {\text{I}}_{\text{p,max}}\cdot \text{K}}\cdot \frac{1}{\left[\text{DRUG}\right]}$$ (1.9)

The binding constant (*K*) between the compounds and CT-DNA, 1.13×10^6^ M^-1^ and 5.35 ×10^5^ M^-1^ for compound I and II respectively, was calculated according to the equation (1.9). These values were typical for metal compounds that bind to DNA via intercalation mode [25]. The binding constant of compound I was about 2.1 times larger than that of compound II, which indicated that the CT-DNA-binding strength of compound I was stronger than that of compound II. A stronger bonding indicated a more stable combination with CT-DNA and thus a better anticancer activity [26].

### 3. Ultraviolet-visible absorbance spectra

Generally, the DNA double-helix structural change, which is caused by its binding with other molecules, may be revealed in spectral features of hyperchromism (rise in absorption intensity) and hypochromism (fall in absorption intensity). The hyperchromism arises from disassembling of DNA double strand [27–28] upon molecular contact, and has been observed frequently in the interaction of DNA with porphyrins, phenanthroliens and salophen complex [29]. In contrast, the hypochromism is resulted from the tightenning of DNA duplex assembly [30–32].

**Fig 6:**
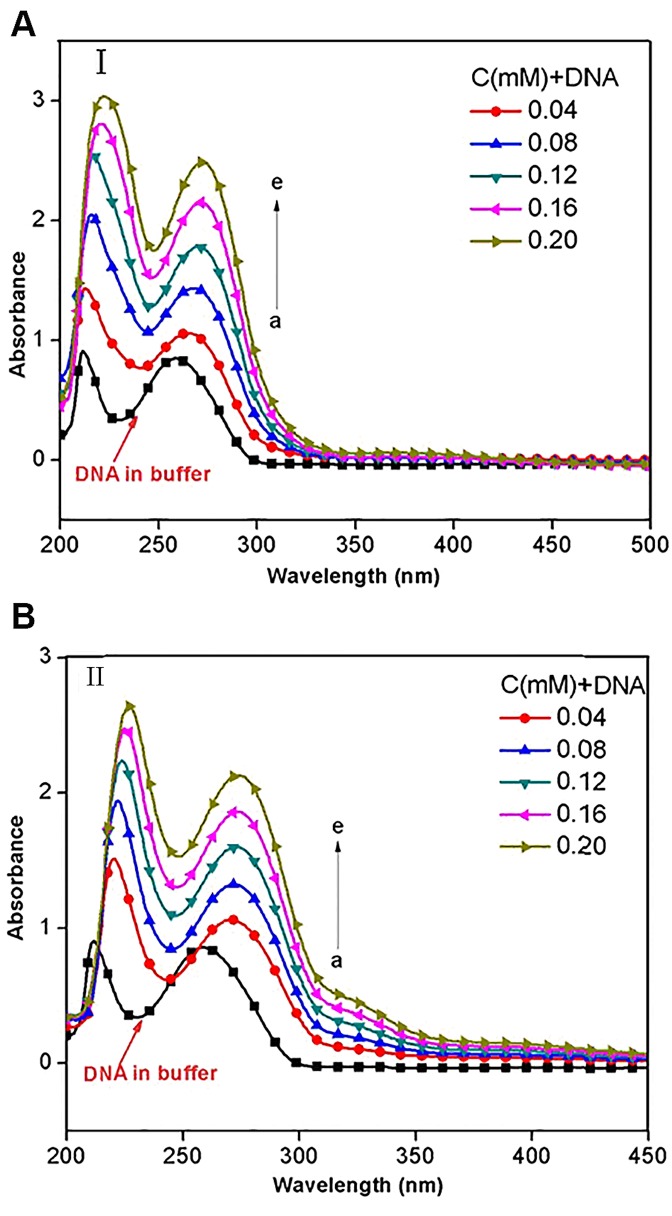
UV absorption spectra of CT-DNA (0.02mM) with various concentrations of compound I (A) and II (B).

In this study, UV-vis absorption spectra were recorded while increasing amount of the two compounds were added respectively to 0.02 mM CT-DNA solution. As shown in Fig. 6A, the maximum absorption of free CT-DNA was at 212 and 260 nm, and the absorption bands showed hyperchromism and a large red shift in *λ*_*max*_ upon the addition of different concentrations of compound **I**. When the compound **I** concentration reached 0.08 mM, the hyperchromism was 49.4% and the red shift was 14–16 nm from band 260 nm. In contrast, as shown in Fig. 6B, the hyperchromism of 53.5% and the red shift of about 13 nm from band 260 nm were observed for compound **II**.

The hyperchromism appearing in absorbance around 258 nm without any hypsochromic effect, but with a bathochromic shift, can be attributed to the breaking of hydrogen bonds between complementary DNA strands, which resulting from intercalations of DNA with small molecules that leads the opening of DNA double helix [33–34]. The present results indicated the existence of strong intercalation of CT-DNA with the two compounds, this was consistent with a typical berberine-DNA intercalation which caused a hyperchromism of 35% with a red shift about 15 nm [35]. Further, the red shift might be ascribed to the intercalation of CT-DNA base pairs with aromatic chromophores of cymene and pyridyl [36–37], due to the decrease in energy gap between the highest and the lowest molecular orbitals (HUMO and LUMO) after CT-DNA binding with the compounds [38].

## Conclusions

Cyclic voltammetry showed that the two compounds, [(*η* ^6^-*p*-cymene)Ru(L1)Cl_2_ and (*η* ^6^-*p*-cymene)Ru(L1)Cl_2_], were adsorbed by CT-DNA which was coated on gold electrode surface, the adsorption behavior was fit for *Langmuir* equation. Further, the hyperchromism of ultraviolet-visible absorbance spectra revealed the nature of the adsorption was intercalation of the two half-sandwich ruthenium(II) compounds with CT-DNA. The binding constant of compound I was approximately 2.1 times larger than that of compound II, which indicated that the compound I showed stronger CT-DNA binding ability than compound II. It might be expected boldly that compound I was a better antitumor agent than compound II.

## Supporting Information

S1 Fig The infrared spectrum of compound I. (TIF)

S2 Fig The ^1^H-NMR spectrum of compound I. (TIF)

S3 Fig The ^13^C-NMR spectrum of compound I. (TIF)

S4 Fig The infrared spectrum of compound II. (TIF)

S5 Fig The ^1^H-NMR spectrum of compound II. (TIF)

S6 Fig The ^13^C-NMR spectrum of compound II. (TIF)
