# Supplementary figures and images for: Two Half-Sandwiched Ruthenium (II) Compounds Containing 5-Fluorouracil Derivatives: Synthesis and Study of DNA Intercalation
Source: PLoS One. 2015 Mar 19;10(3):e0120211. doi: 10.1371/journal.pone.0120211 (PMC4366203; doi:10.1371/journal.pone.0120211)

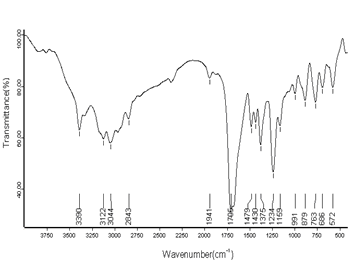

Supplement: S1 Fig — (TIF) [file pone.0120211.s001.tif]

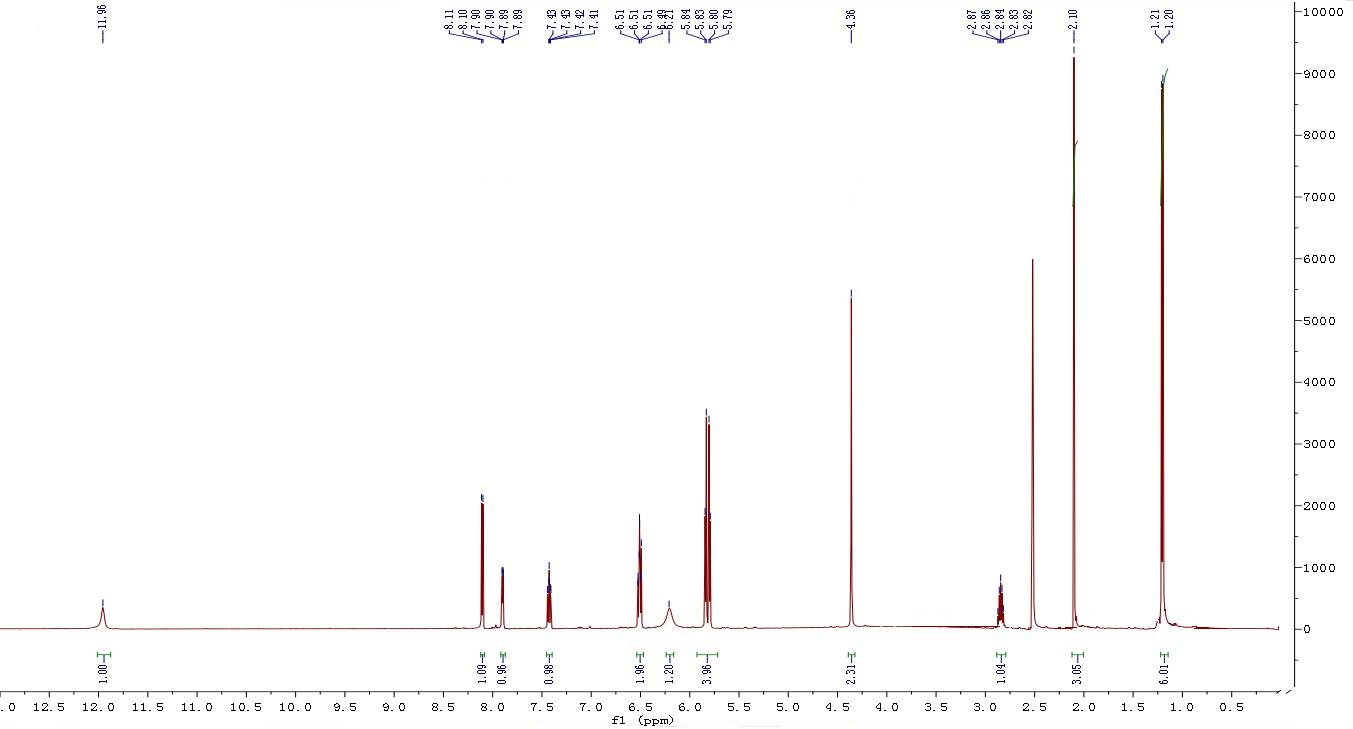

Supplement: S2 Fig — (TIF) [file pone.0120211.s002.tif]

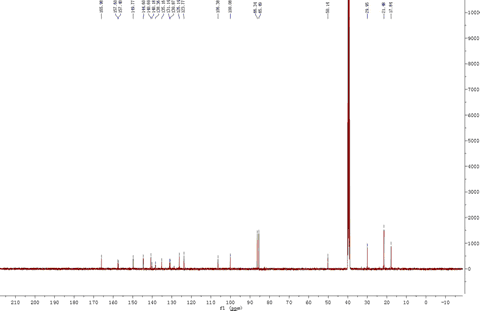

Supplement: S3 Fig — (TIF) [file pone.0120211.s003.tif]

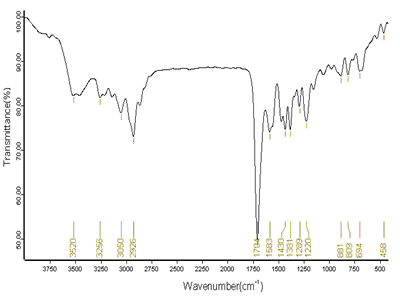

Supplement: S4 Fig — (TIF) [file pone.0120211.s004.tif]

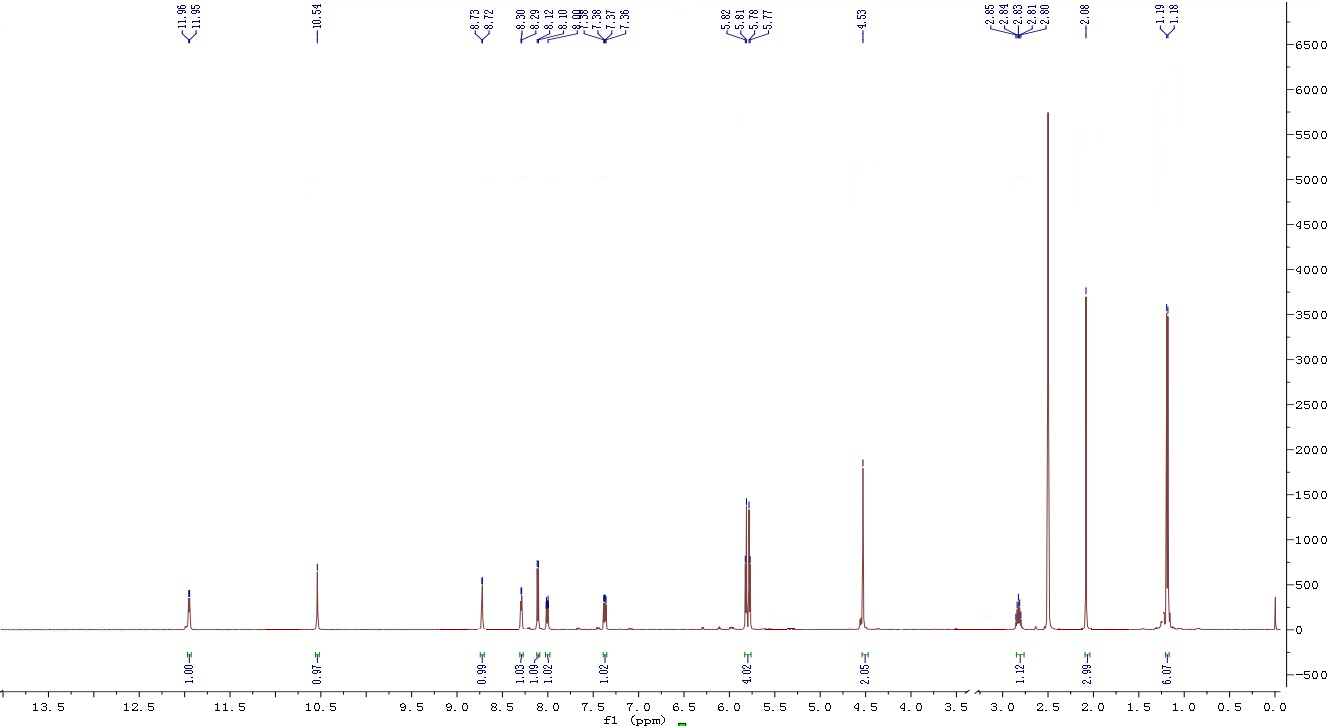

Supplement: S5 Fig — (TIF) [file pone.0120211.s005.tif]

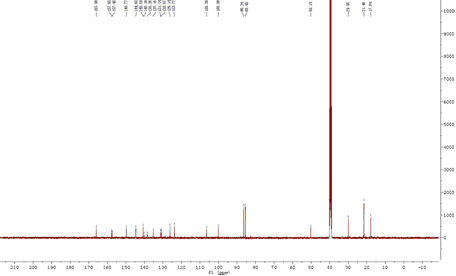

Supplement: S6 Fig — (TIF) [file pone.0120211.s006.tif]
